# Impedimetric Detection and Electromediated Apoptosis of Vascular Smooth Muscle Using Microfabricated Biosensors for Diagnosis and Therapeutic Intervention in Cardiovascular Diseases

**DOI:** 10.1002/advs.201902999

**Published:** 2020-07-27

**Authors:** Anubhav Bussooa, Daniel Hoare, Mahmut T. Kirimi, Srinjoy Mitra, Nosrat Mirzai, Steve L. Neale, John R. Mercer

**Affiliations:** ^1^ BHF Cardiovascular Research Centre University of Glasgow Glasgow G12 8TA UK; ^2^ Scottish Microelectronics Centre Kings Buildings Campus University of Edinburgh Edinburgh EH9 3FF Scotland; ^3^ Bioelectronics Unit University of Glasgow Glasgow G12 8TA UK; ^4^ James Watt School of Engineering University of Glasgow Glasgow G12 8QQ UK

**Keywords:** apoptosis, atherosclerosis, cardiovascular disease, impedance sensors, smart stents

## Abstract

Cardiovascular diseases remain a significant global burden with 1‐in‐3 of all deaths attributable to the consequences of the disease. The main cause is blocked arteries which often remain undetected. Implantable medical devices (IMDs) such as stents and grafts are often used to reopen vessels but over time these too will re‐block. A vascular biosensor is developed that can report on cellularity and is amenable to being mounted on a stent or graft for remote reporting. Moreover, the device is designed to also receive currents that can induce a controlled form of cell death, apoptosis. A combined diagnostic and therapeutic biosensor would be transformational for the treatment of vascular diseases such as atherosclerosis and central line access. In this work, a cell sensing and cell apoptosing system based on the same interdigitated electrodes (IDEs) is developed. It is shown that the device is scalable and that by miniaturizing the IDEs, the detection sensitivity is increased. Apoptosis of vascular smooth muscle cells is monitored using continuous impedance measurements at a frequency of 10 kHz and rates of cell death are tracked using fluorescent dyes and live cell imaging.

## Graphical abstract

Vascular stenosis is a major complication of cardiovascular pathologies such as heart attack, stroke, and failed vascular access. An implantable self‐reporting biosensor that detects the pathological changes offers a diagnostic early warning system. Importantly the sensor has dual function and is able to induce a therapeutic and controlled form of cell death. The design is scalable, with detection sensitivity increasing with miniaturization.

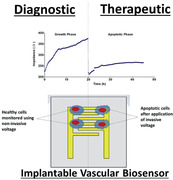

## 1 Introduction

Cardiovascular disease (CVD) has the highest mortality rate in the world, with 1 in 3 deaths attributable to the disease.^[^ ^1^ ^]^ CVD is an umbrella terms that covers a range of pathologies including hypertension, myocardial infarction, cardiac arrest and stroke. Blocked arteries are one of the main precursors for these events. The underlying pathophysiology of arterial disease is caused by vascular smooth muscle cells remodeling and blocking the vessel in response to known risk factors such as smoking, excess alcohol, a sedentary lifestyle and a high fat diet. The resulting fibro‐fatty plaque is termed atherosclerosis.^[^ ^2^ ^]^ In the heart, symptoms often include chest pain such as angina pectoris caused by restriction or occlusion of blood flow within the coronary arteries of the heart. The narrowing of the artery limits the blood flow and oxygen delivery to the heart is diminished.^[^ ^3^ ^]^ There are two major ways of intervening on the vessel stenosis: costly coronary artery bypass grafting (CABG)^[^ ^4^, ^5^ ^]^ and the widespread deployment of intravascular stents termed percutaneous coronary intervention (PCI) or angioplasty.

### 1.1 The Problem of In‐Stent Restenosis

**Figure 1:**
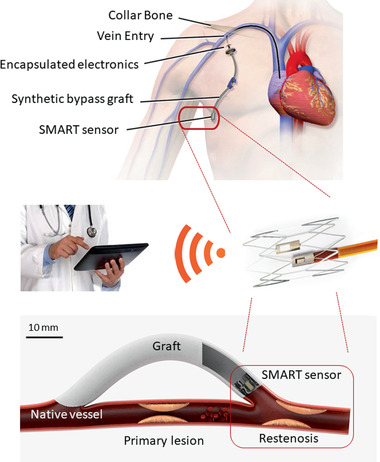
Concept diagram of sensor integrated onto an implantable medical device. The smart sensor is deployed on a synthetic graft inside the distal vein or artery (circled) to bypass a failed segment of vessel. The Smart impedance device is connected to a wireless telemetry unit that reports cellularity, pressure, and flow to a treating clinician through an iOS/Android interface (Top image courtesy of the U.S. National Library of Medicine (D002405); reproduced with permission).

PCI involves balloon angioplasty to reopen by stenting of the culprit vessel but initiates a range of acute reactions including denuding of the inner endothelial layer and tearing of the medial muscular layer, which eventually leads to neointimal regrowth and re‐blocking of the artery as part of the healing response to injury.^[^ ^6^ ^]^ Activation of smooth muscle cell (SMC) proliferation and migration, leads to intimal wall thickening often with progression to restenosis.^[^ ^7^ ^]^ Further work has shown that restenosis has become a common and sometimes fatal consequence whenever stents or grafts are placed in the human body. Drug eluting stents (DES) have significantly reduced, but not eliminated, neointimal proliferation and restenosis for coronary artery stents, through the suppression of SMCs proliferation and migration. Yet their major shortcomings of silent late in‐stent occlusion and thrombosis remains.^[^ ^6^ ^]^ With over 2 million PCI's performed in the USA and Europe alone there is still a clear unmet clinical need to detect these events. Increasingly, synthetic grafts are being used to bypass disease segments of vessels. Wherever a synthetic and native vessel join, a similar wound response occurs that can silently re‐block the vessel (**Figure** **1**).

Anti‐proliferative drugs such as Rapamycin (Sirolimus) are frequently coated on stents and can be classified as either cytostatic or cytotoxic drugs. These inert drug eluting stents (DES) have been very successful but also have limitations. The polymer used to load the drugs is associated with local inflammatory reactions and will non‐specifically elute off the stent making them an effective solution. In up to 10% of cases restenosis will still form and be completely undetected. An alternative approach would be to use an on‐stent biosensor for detection of proliferation of cells and detection of clot, then use local delivery of electroporation to induce a controlled form of cell death termed apoptosis to reopen the vessel.

### 1.2 Electromediated Cells Sensing and Cell Death

The pioneering work of Nobel Laurate Ivar Giaever and Charles Keese established impedimetric cell sensing (Giaever and Keese, 1993). Ample evidence suggests that changes in electrical impedance can be used to detect increased cellularity as the cells act as insulators to the flow of electrical current. Mamouni and Yang (2011)^[^ ^8^ ^]^ carried cell sensing experiments using IDEs. The IDE consisted of 50 pairs of fingers, with 15 µm finger width and 15 µm finger separation. In the absence of cells, and with culture medium only, the impedance of the system comes from two components, which are the resistance of the solution and the double layer capacitance of the electrodes. In the presence of cells on the electrode surface, the equivalent circuit contains the impedance contributions from the cells in addition to the resistance of the solution and double layer capacitance. Equally, electroporation, which is a method of increasing the permeability of the cell plasma membrane by passing an electric field across it, is not new and often used in the lab but when miniaturized becomes an appropriate method for mediating a controlled form of cell death^[^ ^9^ ^]^ for the next generation of implantable medical devices (IMDs). Indeed Giaever went on to establish Applied Biosystems to commercialize the electric cell–substrate impedance sensing system (ECIS) (ECIS is a trademark of Applied BioPhysics Inc.).

Electroporation can be achieved in a transient state, where reversible hydrophilic pores are created in the cell membrane due to changes in the phospholipids in the lipid bilayer^[^ ^10^ ^]^ or in permanent state through the formation of nanopores which remain open.^[^ ^11^ ^]^ This allows for the insertion of fluorophores, drugs, DNA, RNA, tracers, antibodies, ions, or other molecules to the cell. Although this is performed in controlled conditions, some nanopores in the membrane do not always seal after the electrical field has been removed and cell death occurs.

Natural cell death has been identified to involve several pathways and has a number of characteristics. In its simplest form it can be categorised into two broad types, necrosis and apoptosis.^[^ ^12^ ^]^ Apoptosis the controlled and regulated cell death, with characteristic identifiers of cell membrane blebbing, phosphatidylserine flipping, cell and nucleus shrinking, decreased mitochondria permeability, release of cytochrome c and fragmentation of DNA.^[^ ^13^, ^14^ ^]^ In contrast necrosis is an uncontrolled process that displays no distinctive features of apoptosis nor phagocytosis and lysosomal digestion^[^ ^15^ ^]^ and would be predicted to be dangerously thrombogenic.

Piñero et al. (1997)^[^ ^16^ ^]^ carried out an electroporation experiment using HL60 human promyeloid leukemia cells to determine whether cell death was induced by apoptosis, necrosis or both. They used a mixture of three fluorescent dyes: propidium iodide (PI), fluorescein diacetate (FDA) and Hoeschst 33342. Normal living cells showed green (FDA) fluorescence in the cytoplasm and blue (Hoeschst) fluorescence in the nucleus, as both fluorochromes can pass through intact membranes. Cells which died either of apoptosis or necrosis, show DNA stained with red fluorochromes (PI). Apoptotic cells show green cytoplasm with spotted blue‐stained nuclear bodies.

For many cell membranes electropermeabilization is triggered by a threshold potential difference of 200 mV across the membrane and is reversible.^[^ ^17^ ^]^ As such the size of a cell can become important, as larger cells will experience a greater potential difference across them within the same electrical field as a smaller cell^[^ ^18^ ^]^ and it has been shown that the shape of the cell can also become important.^[^ ^19^ ^]^ When electroporation is carried out using short high‐voltage pulses, temporary increase in membrane permeability can be used to enhance the transport of calcium. Calcium is found intracellularly in smooth muscle cells at < 100 × 10^−9^ m while extracellular calcium concentration is ≈1.2 × 10^−3^ m.^[^ ^20^ ^]^ Variations in calcium homeostasis can lead to apoptotic cell death.^[^ ^10^ ^]^ This is due to increased cell membrane permeability from electroporation increases the intracellular calcium levels, which in turn lead to effects such as cessation of ATP production, cell blebbing, destruction of cytoskeleton and cell swelling. Electroporation combined with calcium ions is a promising anticancer therapy which is safe. Kinio and Mills (2017) developed a microfluidic device with interdigitated electrodes (IDEs) printed on the bottom and top of a microfluidic channel. Human blood containing MCF7 cancer cells was flowed through the channel. The interdigitated electrodes allowed fractionation of the blood cells, with cancer cells closer to the top of channel and blood cells closer to the floor of the channel. The electric field closer to the top of the channel was strong enough to cause irreversible electroporation and cell death. Recently, Li et al. (2017)^[^ ^21^ ^]^ studied electroporation of single cells using AC voltages. They fabricated a microfluidic device with co‐planar gold electrodes with a separation of 10 µm. This gap allowed trapping of a single Jurkat cell when a suspension of these cells was streamed through the microfluidic channel. Membrane poration was assessed using propidium iodide (PI) and cell viability was assessed using Calcein AM. Using frequencies of 100 kHz and 10 MHz cell electroporation was identified at 0.6 V at 100 kHz and 2.3 V at 10 MHz. A decrease in cell viability at 1.0 V for 100 kHz and 2.7 V for 10 MHz resulted in cell death at increased voltages to 1.3 V for 100 kHz and at 4.2 V for 10 MHz.

Stolwijk, Michaelis, and Wegener (2012)^[^ ^22^ ^]^ provide precedence for this approach by studying the effects of applying an invasive sinusoidal voltage pulse of 5 V and 40 kHz which was applied for 30 s to normal rat kidney (NRK) cells using ECIS electrodes. The cells were stained using a live–dead stain (calcein acetoxymethyl (CAM) and ethidium homodimer (ETHD)). The control well showed only green fluorescence (CAM) indicating that all the cells were healthy. The treatment well showed an area of red fluorescence (ETHD) surrounded by an area of green fluorescence, indicating that cells directly on the electrodes were dead, while cells around the electrode were unaffected. Their work shows that application of the pulse to the treatment well caused a drop in normalized impedance at 400, 4000, and 40000 Hz.

Herein, we report the use of a similar microfluidic device using interdigitated electrodes to monitor health of a primary smooth muscle cell monolayer and to induce apoptosis of these cells using low pulse doses of alternating currents (AC). In the future we propose in our recent reviews how these could be integrated into existing implantable vascular devices.^[^ ^23^, ^24^ ^]^

## 2 Experimental Section

**Figure 2:**
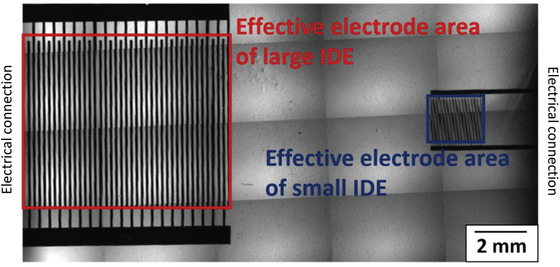
Multiple image alignment (MIA) micrograph of large IDE and small IDE with red and blue boxes, respectively showing effective electrode area.

Interdigitated electrodes (IDEs) were fabricated at the James Watt Nanofabrication Centre of University of Glasgow using standard microfabrication techniques (**Figure** **2**). The photolithographic mask used had two different sizes of IDE. The large IDE (4 mm^2^) consisted of two sets of 20 fingers (length 800 µm, width 100 µm, and 100 µm separation between each finger). The small IDE (0.5 mm^2^) consisted of two sets of 20 fingers (length 200 µm, width 25 µm, and 25 µm separation between each finger). The electrodes were patterned on a microscope slide (Sigma Aldrich, UK) with a 10 nm titanium adhesion layer and 100 nm gold layer. The photolithographic mask used had two different sizes of IDE (Figure 2). A plastic chamber from a commercially available slide chamber (Sigma Aldrich, UK) was mounted onto the patterned glass slide using UV curable glue (Loctite, Germany). Electrical wires were soldered to the contact pads of the small IDE.

### 2.1 Experimental Setup

The experimental setup consisted of a Live cell microscope (Olympus IX71, Japan) with a temperature and CO_2_ controlled enclosure (Solent Scientific, UK), an LCR meter (Hioki IM 3536, Japan), a function generator (AimTTi – TG5011A, UK) and a PC for interfacing with the microscope and the LCR meter. The IDEs were connected to the LCR meter for impedance measurements and to the function generator for applying controlled voltages for electroporation.

### 2.2 Primary Cell Seeding for Impedance Measurements

The fabricated device was sterilized by misting with 70% ethanol and washing with deionized water. The baseline impedances were first measured with culture medium only. An LCR meter (Hioki IM3536) with a fixed current, varying voltage system was used for measuring impedance with a constant current (CC) setting of 10 µA. The impedances were measured at 10, 20, 30, 40, and 50 kHz using an LCR meter for the large IDE and small IDE. A frequency range of 10–40 kHz was recommended by Wang et al. (2008)^[^ ^25^ ^]^ for maximum sensitivity when monitoring cells using IDE. An adherent primary vascular cell type, mouse aortic smooth muscle cells (MASMCs), previously characterized,^[^ ^26^ ^]^ were used for all experiments. The cells were cultured in phenol red free DMEM media supplemented with 10% fetal bovine serum (FBS) in cell culture flasks. Once sub‐confluent, the cells were trypsinized and 200 000 MASMCs were then seeded into the fabricated device, and the cell suspension was pipetted a few times to ensure a homogeneous population was plated. MASMCs were spherical when in suspension and spindle‐shaped, once adhered to the culture substrate. The cells were allowed to settle down for 18 h inside an incubator (37 °C and 5% CO_2_) to ensure that they adhered to the electrodes. Experimental impedances were then recorded. The increases in impedance were calculated at each frequency for the large and small IDEs.

### 2.3 Impedance Measurements, Time‐Lapse Live Cell Imaging, and Induction of Cell Death

**Figure 3:**
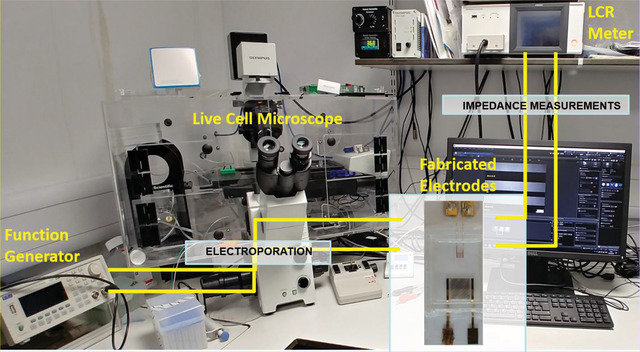
Live cell microscope with a temperature and CO_2_ controlled enclosure, an LCR meter, a function generator and a PC for interfacing with the microscope and the LCR meter.

Primary MASMCs were seeded into sterilized fabricated devices (200 000 cells per mL) and grown in phenol red free DMEM media. The devices were transferred to the microscope stage with warmed (37 °C) and CO_2_ buffered enclosure and the large IDE was connected to the LCR meter. Bright field imaging and impedance measurements (at 10 kHz) were simultaneously started and acquired at 15 min intervals. At the 20 h time point, the LCR meter was disconnected from the electrodes, and the function generator (AimTTi – TG5011A) was connected so as to apply a sine voltage of 2 V_peak‐to‐peak_ 40 kHz for 2 min, the minimum duration required to observe a drop in impedance (**Figure** **3**). To clarify the impedance readings were independent of the applied voltage from the function generator. Equally changes in impedance after voltages were applied were directly due to changes in the cellularity across the sensor surface. The function generator was then disconnected and the LCR meter reconnected so as to continue impedance measurements up to the 45 h timepoint and comparisons made between the small IDE and large IDE. To provide a positive control for comparing the effects of electromediated cell death, the chemotherapeutic drug etoposide and a mechanical scratch were used. Etoposide is a chemotherapy drug used to treat cancer. It prevents cell proliferation by inhibiting a class of DNA repair enzymes called Topoisomerase II.^[^ ^27^ ^]^ Its use in the study was purely to act as a drug based positive control to induce the controlled form of cell death apoptosis. Equally the mechanical scratch assay was a physical insult to the cell monolayer that would induce both uncontrolled necrotic and apoptotic death.

### 2.4 Fluorescent Visualizing Apoptotic Cell Death

200 000 MASMCs were seeded (0 h timepoint of experiment) into a fabricated device. 2 mL of Phenol‐red‐free DMEM was added. The cells were allowed to settle and adhere to the bottom of the device for 23 h inside the microscope enclosure, with impedance measurement acquired at 10 min intervals. At 23 h, the cells were stained using the Apoptosis/Necrosis Assay Kit (ab176749 – Abcam, UK). The kit was a mixture of three dyes, CytoCalcein Violet 450, Apopxin Green Indicator and 7‐AAD for detecting live, apoptotic and necrotic cells respectively. The staining solution was prepared by pipetting 5 µL of each of the three dyes into 500 µL of Phenol‐red‐free serum‐free DMEM. The cell monolayer was carefully washed twice using Phenol‐red‐free serum‐free DMEM. Then the staining solution was pipetted onto the monolayer and the dyes were allowed to incubate for 1 h. Poststaining the staining solution was pipetted out of the chamber and the monolayer was washed twice using Phenol‐red‐free DMEM. Then 2 mL of fresh Phenol‐red‐free DMEM was pipetted into the chamber which was transferred to the microscope stage.

### 2.5 Poststaining Monitoring and Application of Voltage

Microscopic images were acquired under bright field (BF) and in the DAPI, FITC, and Texas Red fluorescent channels. This was started at the same time as impedance measurements, and both were acquired at 10 min intervals. The cells were monitored from 24 h (immediately after staining) to 32 h to ensure that the staining was not affecting cell viability. At 32 h, the chamber was disconnected from the LCR meter and connected to a function generator. A sine voltage of 2 V peak‐to‐peak 40 kHz was applied for 2 min, the minimum duration required to observe a drop in impedance. The chamber was then reconnected to the LCR meter and impedance measurements resumed. The impedance and microscopic images were acquired up to the 49 h timepoint. Timelapse imaging and impedance measurements were stopped, and the cell chamber was transferred to a cell culture hood again. The staining was carried out again, in exactly the same way as before. After the 1 h staining step, the cell chamber was transferred to the live cell microscope again, and final BF and fluorescent images of the cells were acquired.

## 3 Data Collection and Statistical Analysis

Impedance data were collected using a Hioki LCR meter IM3536 with technical replicates made using intermittent measurements, as the meter probes could be disconnected and connected to different set of electrodes between measurements. For continuous measurements the probes remained connected to only one electrode at a time. All data presented are representative of replicate data samples with as mean ± standard deviation (SD). *P* values were calculated using student's paired *t*‐tests (two‐tailed distribution) when comparing two groups, one way analysis of variance (ANOVA) was used to determine significant difference between two or more independent groups, with a Tukey post‐test for large sample sizes. Statistical significance was displayed as *P* < 0.05 (one star), *P* < 0.01 (two stars), or *P* < 0.001 (three stars). All analysis was performed and plotted on Graph Pad Prism (v 5).

## 4 Results

**Figure 4:**
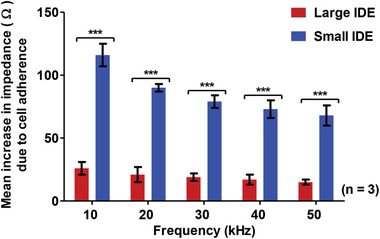
Mean increases in impedance at different frequencies due to 200 000 MASMCs detected by large IDE and small IDE. Two‐way ANOVA showed that there was a statistically significant (*p* < 0.001) difference in the mean increase in impedance across all the frequencies (10–50 kHz) *n* = 3.

The results from **Figure** **4** and **Table** **1** show that the mean increase in impedance was higher with the small IDE as compared to the large IDE at 10, 20, 30, 40, and 50 kHz. Optimum sensing frequency range was previously determined from 10–50 kHz (Figure S1, Supporting Information). Two‐way ANOVA showed the mean increases with small IDE and large IDE were significantly different (*p* < 0.001) at all the frequencies. The highest increase in impedance was detected at 10 kHz for both the small IDE and large IDE. The small IDE had an effective electrode area 16 times smaller than the effective electrode area of the large IDE. The ratio of the mean increase with small IDE over large IDE had a mean value of 4.35. Importantly this suggests the relationship between mean increase in impedance detected due to MASMCs is inversely proportional to the electrode area.

**Table 1:** Comparison of increases in impedance at the different frequencies for large

Frequency [kHz]	[A] Impedance increase [Ω] with large IDE	[B] Impedance increase [Ω] with small IDE	Ratio $\frac{B}{A}$
10	26	116	4.46
20	21	90	4.29
30	19	79	4.16
40	17	73	4.29
50	15	68	4.53
Mean			4.35

**Figure 5:**
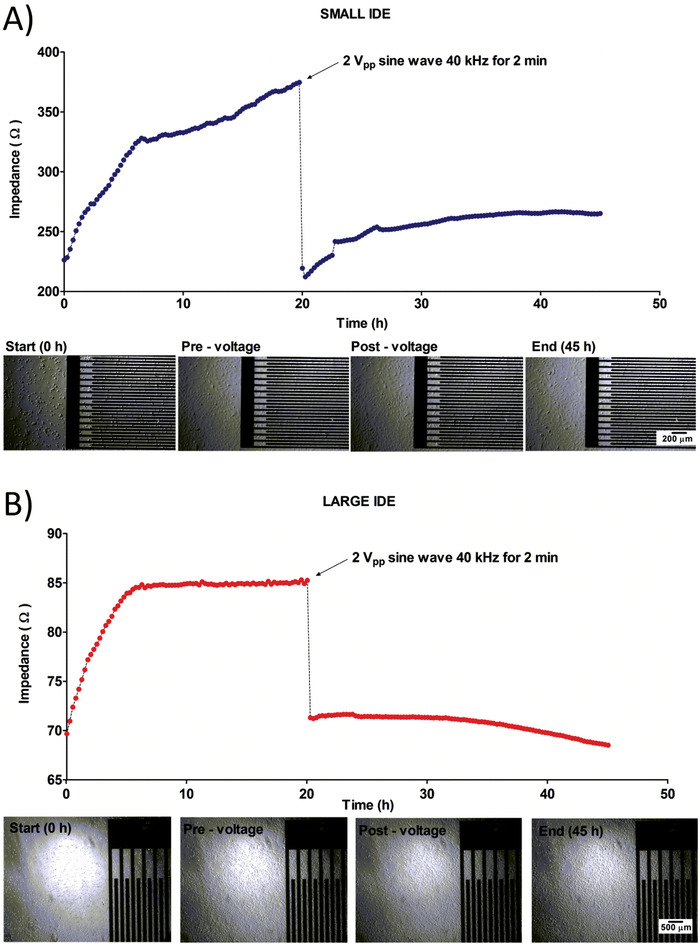
A,B) Detection of cell adherence over time and induction of cell death by comparison of impedance measurements of large IDE (A) and small IDE (B) performed over 45 h with a pulse voltage for 2 min applied at 20 h to induce electroporation.

**Figure** **5** shows that both the large IDE and small IDE are able to track gradual adherence of MASMCs to the electrodes sensor. The large IDE detected an increase of ≈15 Ω when the cells had settled down while the small IDE detected a maximum increase of ≈150 Ω. After the application of the voltage, the impedance dramatically dropped by 15 Ω for the large IDE and by 160 Ω for the small IDE. In both cases, the impedance dropped approximately back to the baseline impedance at 0 h. Following this drop, the impedance did not go back up to the pre‐voltage impedance, implying that the voltage causes a permanent effect on the cells. However, any physical effect across the on the cells were not immediately observable using bright‐field microscopy (BFM). Moreover, the effects seemed to be restricted to the regions in‐between the electrode fingers rather than on the fingers. The cells around the IDE appeared mostly unaffected by the application of the voltage. This implied that a very specific and localized effect can be applied to a cell monolayer.

**Figure 6:**
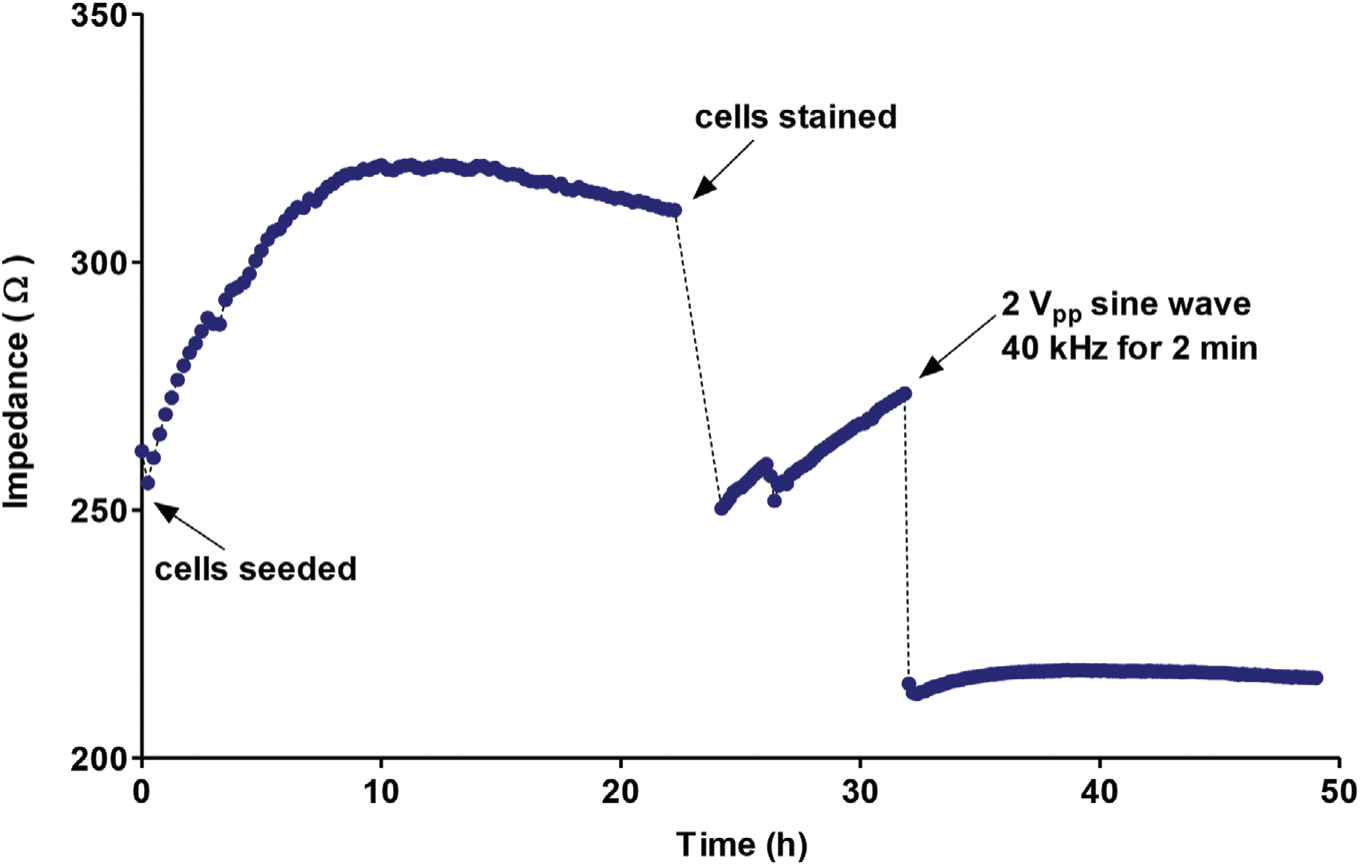
Impedance measurement for the whole experiment showing impedance immediately postseeding, during cell adherence, poststaining, and postvoltage.

**Figure** **6** shows the impedance readings for the whole duration of the experiment (49 h). The baseline impedance at 0 h was 260 Ω. The impedance reached a maximum of 320 Ω at 10 h and gradually decreased to 310 Ω at 22 h. This could be attributable to a depletion of the amount of nutrients in the media, causing the cells to detach slightly from the electrodes and establishes the current maximum duration for these types of experiments. At 22 h, the impedance measurements were stopped and the staining step was carried out. The impedance measurements were resumed at 24 h and acquired at 10 min intervals. The staining solution causes the cells to detach slightly from the electrodes, causing the impedance to drop to 250 Ω. The cells were allowed to reattach to the electrodes until 32 h, where an impedance of 273 Ω was reached. The voltage was applied at that point, causing an immediate drop in impedance to 215 Ω. The impedance then stayed relatively constant from this point to 49 h.

**Figure 7:**
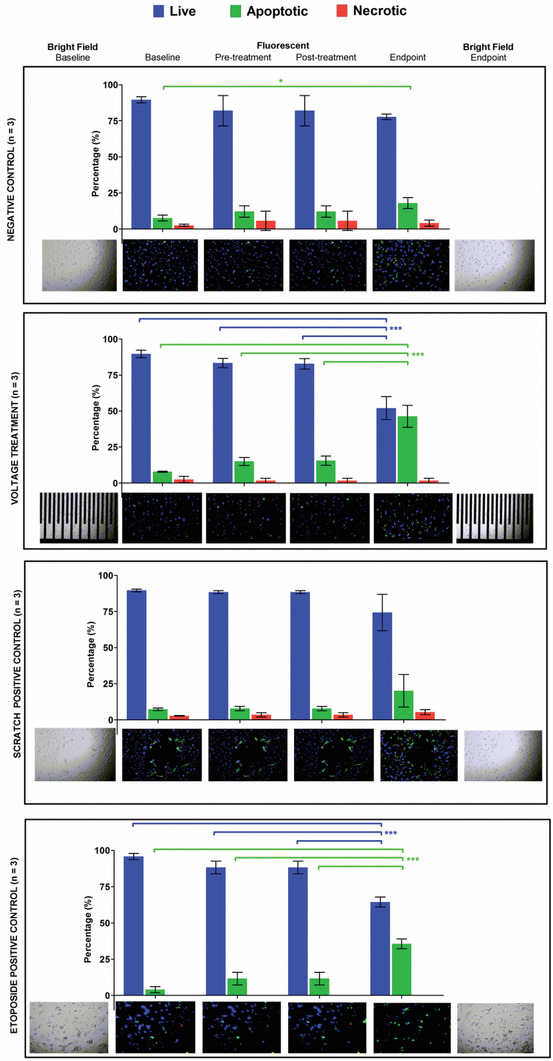
Mean percentages of live, apoptotic, and necrotic cells at the different time points for the negative control without voltage, treatment with voltage, positive control with scratch and positive control with 50 × 10^−6^ m etoposide.

To assess the effects across the monolayer more precisely we used fluorescent live cell imaging. To mimic the effects of mechanical damage that might be expected during stent deployment to the vasculature a scratch assay was compared to a drug induced form of cell death and used as a positive control for the subsequent experiments. **Figure** **7** shows the mean percentages of live, apoptotic and necrotic cells for the negative control, voltage treatment, scratch positive control and etoposide (a topoisomerase inhibitor of DNA repair) as a positive control. The negative control, voltage treatment and scratch positive control were carried out as part of the same experiment, while the etoposide positive control was carried out as part of a separate experiment. The live cell percentage was based on the bright field and blue fluorescence while the apoptotic and necrotic cell percentages were based on the green fluorescence and red fluorescence, respectively.

The percentages were compared using one‐way ANOVA with Tukey's multiple comparison posthoc test. For the negative control, the percentage of apoptotic cells at the endpoint was statistically different (*p* < 0.05) from the baseline, implying that there was an important increase in apoptosis from 8% to 18%. Comparing the pre‐voltage treatment at baseline to the etoposide drug positive control post‐treatment highlighted a statistically difference in the percentages of live and apoptotic cells by the endpoint (*p* < 0.001 and *p* < 0.001, respectively). This implies that the induction of apoptosis using 2 V sine wave for 2 min had similar effects to 50 × 10^−6^ m etoposide at 24 h. The percentage of apoptosis induced by the voltage was higher (46% vs 36%) compared to etoposide. Hence, a short pulse of voltage can induce a higher percentage apoptosis in a MASMC monolayer compared to a prolonged exposure to etoposide.

## 5 Conclusions

Intravascular biosensors have the potential to transform cardiovascular healthcare. A number of groups are actively researching cell sensing technologies but few, if any, are including therapeutic induction of cell death.^[^ ^28^, ^29^, ^30^ ^]^ Here we have demonstrated that we were able to develop a cell sensing and cell apoptosing system based on the same interdigitated electrodes. Moreover, our sensor is entirely scalable to virtually any size. Miniaturizing the effective electrode area of the interdigitated electrode by a factor of 16 (linear factor of 4) increased the sensitivity of the cell detection approximately by a factor of 4.35 over the 10–50 kHz frequency range. These results are in line with findings by Zhang et al. (2017),^[^ ^31^ ^]^ who obtained an increases in sensitivity by factors of 1.7 and 1.5 (at different cell densities), when miniaturizing their electrodes by a linear factor of 1.5. All measurements in this study were carried out at 8 kHz, which was the optimum sensing frequency for their electrode design. We compared the increases in impedance at several frequencies. Results from Figure 4 show that the maximum impedance increases were obtained at 10 kHz for both small IDE and large IDE. Thus this frequency was chosen for continuously monitoring cell adherence, proliferation and apoptosis. Montaño‐Figueroa et al. (2019)^[^ ^32^ ^]^ also used 10 kHz to monitor apoptosis. However, in these experiments apoptosis was chemically induced, while in our experiments, apoptosis was electrically induced. By applying a sine wave of 2 V at 40 kHz for 2 min, the same electrodes could not only be used for cell detection but importantly also be used to induce apoptosis, a controlled form of smooth muscle cell death across the sensor surface and beyond the immediate sensing area.

The voltage characteristics in our design were similar to the ones used by Stolwijk, Michaelis, and Wegener (2012) who studied the effects of applying an invasive sinusoidal voltage pulse of 5 V and 40 kHz for 30 s to NRK cells. They observed a clear area of cell death on the electrodes and an area of unaffected cells around the electrodes. They also observed a drop in impedance after application of the invasive voltage. However, they measured the impedance for only a short time period of 0.5 min after voltage application, and it is not clear whether the impedance changes further beyond that point.

In our study, the impedance was measured for ≈25 h after voltage application. During that period, the impedance decreased for the large IDE and increased for the small IDE (without reaching the pre‐voltage‐application impedance).

The ECIS system provides prior art in cell detection that involves the use of electric fields between electrodes to monitor behavior of cells (Ablin and Jiang, 2012). A small alternating current of ≈1 µA is applied through the electrodes, resulting in a small voltage drop of a few millivolts across the cells. From the voltage drop and applied electric current, the impedance can be calculated. Their system is based on the use of specialized culture plates for in vitro evaluation only and is in no way amenable to integration onto implantable medical devices (IMDs). However, the frequency at which the impedance is measured determines the pathway of the electric current, thus allowing different properties such as cell capacitance, barrier function and electrode coverage to be monitored.

Indeed Wang et al. (2008)^[^ ^25^ ^]^ studied the effects of varying these parameters of interdigitated electrode on cell detection sensitivity, and concluded that electrodes were most sensitive to cells in the 10–40 kHz range. In contrast to cell sensing Montaño‐Figueroa et al. (2019)^[^ ^32^ ^]^ used impedance‐base sensors at a single frequency of 10 kHz to monitor the effects of chemically induced apoptosis on pre‐osteoblast cells but not electroinduced cell death. Zhang et al. (2017)^[^ ^31^ ^]^ studied the effects of changing the electrode dimensions on the impedance increase detected but again there is no previous art of the same sensor being used for both cell sensing and cell death that is both scalable and ready for integration into implantable medical devices. In separate experiments, they seeded bovine aortic endothelial cells at densities of 10 000 and 20 000 cells per cm^2^ on working electrodes with radii 150 and 100 µm. When comparing the same cell density, their smaller electrode provided larger impedance increases. This showed that smaller electrode could detect finer changes in cell density in keeping with our findings. The linear size ratio of the large electrode to the small electrode size was 1.5. The ratio of impedance increase of small electrode to large electrode was 1.7 at 10 000 cells per cm^2^ and 1.5 at 20 000 cells per cm^2^. These findings point towards an inverse relation between electrode size and increase in impedance detected that make the design of our electrodes particularly suitable to miniature implantable medical devices.

Indeed, the recovery profiles for the large and small IDEs in our work are different because of the size and sensitivities of the electrodes. The electroporation and subsequent apoptosis are initially localized to the regions within the electrode area, that is on and between the electrode fingers, but the “bystander effect” can extend the region of effect. Fluorescent live cell imaging has allowed us to confirm this and that the cell death induced by the sensor voltage was significantly attributable to apoptosis.

With apoptosis being a much more controlled form of cell death, where the apoptotic bodies are cleared by phagocytes, it offers the hope of a useful system that could be integrated with existing implantable medical devices. Indeed, we have developed a miniaturized and implantable telemetry reporter to integrate with our sensor that can be tested using stentable arteries ex vivo.^[^ ^33^ ^]^ For example, if the cell death and remote cell monitoring using impedance measurements were developed in combination with a vascular device, then a self‐reporting diagnostic and therapeutic cell sensor could be achieved. Diagnosing and assessing cellularity using implantable medical devices is important area of clinical research. Many human pathologies are attributable to inappropriate growth of cells, such as cancers and many vascular pathologies. Future work will now investigate the potential of integrating our design and developing clinically relevant devices.

## Conflict of Interest

The authors declare no conflict of interest.

## Supporting information

Supporting Information
