# Supplementary material for: Impedimetric Detection and Electromediated Apoptosis of Vascular Smooth Muscle Using Microfabricated Biosensors for Diagnosis and Therapeutic Intervention in Cardiovascular Diseases
Source: Adv Sci (Weinh). 2020 Jul 27;7(18):1902999. doi: 10.1002/advs.201902999 (PMC7509665; doi:10.1002/advs.201902999)
Supplement: Supplementary file 1 — Supporting Information [file ADVS-7-1902999-s001.pdf]

Copyright WILEY-VCH Verlag GmbH & Co. KGaA, 69469 Weinheim, Germany, 2020.

ADVANCED SCIENCE
Open Access

Supporting Information

for *Adv. Sci.*, DOI: 10.1002/advs.201902999

Impedimetric Detection and Electromediated Apoptosis of Vascular Smooth Muscle Using Microfabricated Biosensors for Diagnosis and Therapeutic Intervention in Cardiovascular Diseases

*Anubhav Bussooa, Daniel Hoare, Mahmut T. Kirimi, Srinjoy Mitra, Nosrat Mirzai, Steve L. Neale, and John R. Mercer**

Supplemental data

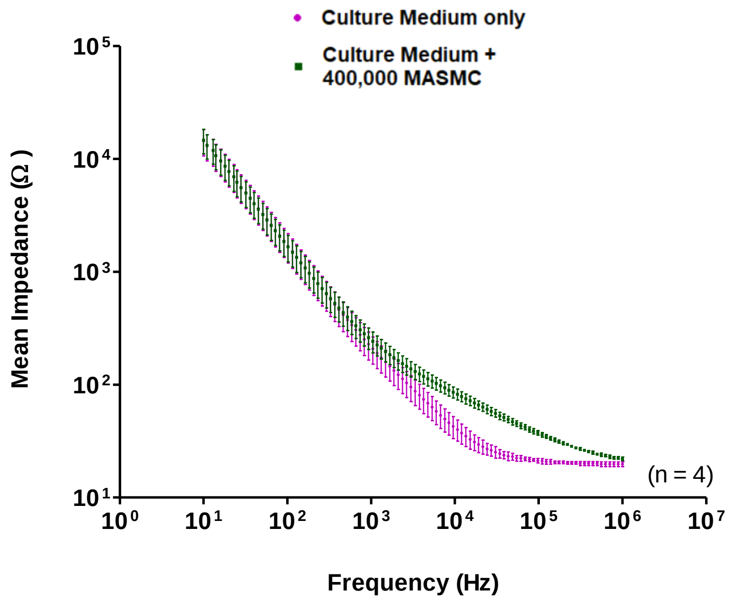

Supplemental Figure 1) Broad frequency sweeps from 10 Hz to 1 MHz to identify optimum sensing frequency range for the fabricated electrodes. The purple data point represents the cell free impedances while the green data points represent the impedance with 400,000 MASMCs seeded.

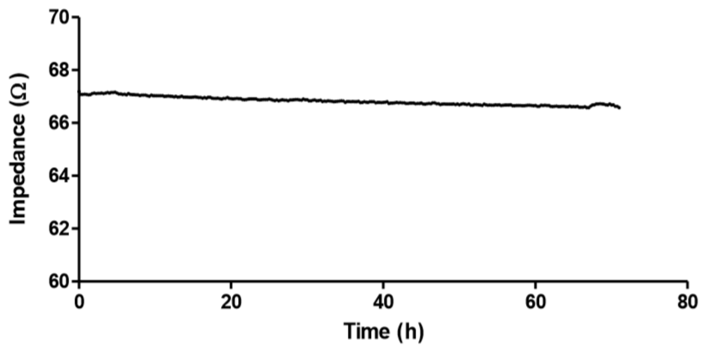

Supplemental Figure 2) Continuous impedance at 10 kHz with normal culture media (DMEM) only without cells. $C0_2$ is used to buffer the media to prevent changes in pH and temperature was kept constant at 37 ^{0}C.

BRIGHT FIELD IMAGE	
**Immediately before staining**	**Immediately after staining**
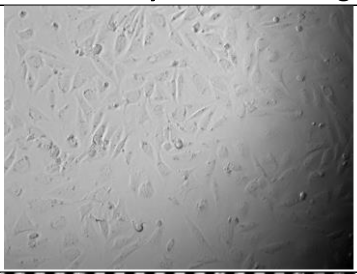	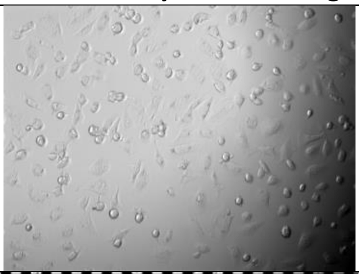
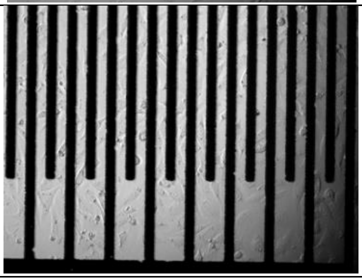	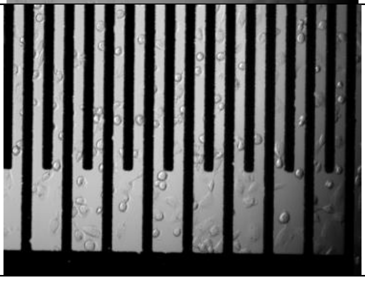

Supplemental Figure 3: Bright field images at one negative control position and one treatment position taken immediately before and immediately after staining.
